# Diagnostic Role of ChatGPT in the Detection of Intracranial Hemorrhage on Non-contrast Computed Tomography: A Retrospective Case-Control Study

**DOI:** 10.7759/cureus.107065

**Published:** 2026-04-14

**Authors:** Suprith J Shankar, Anil K Sakalecha, Goravimakalahalli Srinivasareddy Hemanth Kumar, Prabhakar Kamarthy, Soumya Chincholikar

**Affiliations:** 1 Radiodiagnosis, Sri Devaraj Urs Medical College, Kolar, IND; 2 General Medicine, Sri Devaraj Urs Medical College, Kolar, IND

**Keywords:** ai and machine learning, artificial intelligence in radiology, brain ct scan, chatgpt, diagnostic accuracy study, intracranial hemorrhage, neuroradiology

## Abstract

Background

Intracranial hemorrhage is a potentially fatal neurological emergency. It requires rapid diagnosis to guide the management plan. Non-contrast computed tomography (NCCT) is the primary imaging method for detecting acute intracranial bleeding due to its speed and accessibility. Recent advances in artificial intelligence (AI), including large language models like ChatGPT (OpenAI, San Francisco, CA, USA), offer new opportunities to support radiological interpretation.

Objective

This study evaluated ChatGPT's ability to detect intracranial hemorrhages on NCCT brain images and compared its diagnostic performance with that of radiologists.

Methods

A retrospective case-control study analyzed 276 computed tomography (CT) brain scans obtained from December 2025 to February 2026. The dataset comprised 138 cases with confirmed intracranial hemorrhage and 138 control cases without hemorrhage. CT images were evaluated using ChatGPT with a structured prompt. Radiologist reports served as the reference standard. Diagnostic performance was measured by sensitivity, specificity, accuracy, positive predictive value (PPV), and negative predictive value (NPV). Statistical analyses included chi-squared tests and Cohen's kappa coefficient.

Results

ChatGPT identified 124 of 138 hemorrhage-positive cases and 117 of 138 hemorrhage-negative cases, resulting in 89.9% sensitivity, 84.8% specificity, and 87.3% diagnostic accuracy. Subtype analysis revealed the highest sensitivity for intraparenchymal hemorrhage (88.2%), followed by subarachnoid (73.8%), epidural (66.7%), and subdural (61.5%) hemorrhages, respectively. A statistically significant association was found between ChatGPT predictions and radiologist diagnoses (χ² = 154.1; p < 0.001). The agreement between ChatGPT and radiologist interpretations was good (κ = 0.75). McNemar's test showed no statistically significant difference between ChatGPT and radiologist diagnoses (p = 0.31).

Conclusion

ChatGPT exhibited promising sensitivity in detecting intracranial hemorrhage on NCCT brain scans. However, its moderate specificity suggests it should serve as an adjunct to, rather than a substitute for, radiologist interpretation. Additional research involving larger datasets and model optimization is necessary prior to clinical implementation.

## Introduction

Intracranial hemorrhage is a critical neurological condition linked to significant morbidity and mortality globally. It arises from trauma, hypertension, vascular malformations, aneurysm rupture, or anticoagulant therapy. Early detection is essential for proper clinical management [1].

Non-contrast computed tomography (NCCT) is the preferred investigation for detecting acute intracranial hemorrhage due to its rapid acquisition, widespread availability, and high sensitivity in the detection of acute blood products [2]. Beyond detecting hemorrhage presence, computed tomography (CT) imaging also assesses the location, extent, and related complications, including mass effect and hydrocephalus.

The rapid expansion of imaging data in modern radiology has increased interest in artificial intelligence (AI), particularly large language model (LLM)-based tools designed to assist with image interpretation. Deep learning algorithms have demonstrated high diagnostic accuracy in detecting intracranial hemorrhage on CT imaging and have the potential to enhance workflow efficiency in emergency radiology settings [3-7].

LLM, such as ChatGPT (OpenAI, San Francisco, CA, USA), constitute a recent category of AI systems capable of analyzing complex textual and visual data. These models have been increasingly investigated for applications in healthcare and medical research. With the advancement of multimodal capabilities, they have shown potential in medical image interpretation and clinical decision support [8,9].

Recent studies have examined the application of ChatGPT in radiology. Zhang et al. evaluated GPT-4 for CT image analysis of cerebral hemorrhage, reporting the promising detection of hemorrhagic findings [10]. Similarly, Koyun et al. assessed ChatGPT-4o's diagnostic capability for intracranial hemorrhage on CT scans, demonstrating moderate performance [11].

Specialized deep learning systems have demonstrated strong performance in hemorrhage detection; however, the potential role of general-purpose AI models, such as ChatGPT, remains insufficiently explored. Therefore, the present study aimed to evaluate the diagnostic performance of ChatGPT in detecting intracranial hemorrhage on NCCT brain scans and compare its findings with radiologist interpretations.

## Materials and methods

Study design and population

This retrospective case-control study was conducted at R. L. Jalappa Hospital & Research Centre, Tamaka, Kolar, Karnataka, India (a teaching hospital of Sri Devaraj Urs Medical College, a constituent unit of Sri Devaraj Urs Academy of Higher Education and Research), following institutional approval from the Central Ethics Committee of Sri Devaraj Urs Academy of Higher Education and Research, Kolar (approval number: SDUAHER/R&D/CEC/SDUMC-PG/149/NF/2025-26). Due to the retrospective nature of the study, the requirement for informed consent was waived.

A total of 556 CT brain scans performed during the study period (December 2025 to February 2026) were screened for eligibility. A total of 276 NCCT brain examinations were included in the final analysis, comprising 138 hemorrhage-positive and 138 hemorrhage-negative cases. 

Sample size estimation was performed using Buderer's formula based on previously published studies [8,9]. Assuming an expected sensitivity of 80% and specificity of 60%, with a 95% confidence level and an absolute precision of 6%, the minimum required sample size was calculated to be approximately 235 examinations. The final sample size of 276 exceeded this requirement, enhancing the reliability of the study findings.

Inclusion criteria were patients aged ≥18 years who underwent NCCT brain imaging. Exclusion criteria included postoperative scans, significant motion or beam-hardening artifacts, intracranial mass lesions mimicking hemorrhage, and incomplete imaging data.

The study was conducted in accordance with the Standards for Reporting of Diagnostic Accuracy Studies (STARD) guidelines.

Data collection

Brain CT scans were retrieved from the institutional Picture Archiving and Communication System (PACS) for analysis. Prior to inclusion in the study, all images were anonymized to remove patient identifiers in order to maintain patient confidentiality.

NCCT brain imaging protocol

NCCT brain examinations were performed using a standardized imaging protocol on Siemens SOMATOM go.Top 128-slice scanner (Siemens Healthineers, Erlangen, Germany). Images were acquired in the axial plane from the foramen magnum to the vertex using 120 kV with automated tube current modulation (CARE Dose4D; Siemens Healthineers, Erlangen, Germany). Image reconstruction was performed with 1-mm slice thickness, and images were reviewed using brain parenchymal window settings (window width 80, window level 35) to optimize the visualization of intracranial structures and hemorrhagic lesions.

CT image selection

For each case, 4-6 consecutive axial slices were selected due to upload limitations of 20 MB, ensuring the inclusion of hemorrhagic regions in cases and corresponding anatomical levels in controls. Images were anonymized, standardized, and analyzed in randomized order. To minimize anatomical bias, the overall distribution of anatomical levels in the control group was matched to that of the hemorrhage group, although exact one-to-one matching was not performed. All images were standardized to a minimum resolution of 512 × 512 pixels. Image orientation was verified by radiologists to ensure that no images were flipped, rotated, or mirrored during preprocessing.

All CT images were exported from the PACS in standard image formats (PNG/JPEG) prior to analysis. Images were anonymized to remove patient identifiers. No additional preprocessing, image enhancement, or segmentation was performed apart from the standardization of resolution and orientation. The images were uploaded directly to the ChatGPT interface for analysis.

ChatGPT model and configuration

ChatGPT (GPT-5.3 multimodal model, accessed via the ChatGPT Plus web interface in March 2026) was used for image analysis. All analyses were performed using the default model settings without additional fine-tuning or external training. Each case was analyzed independently in a new chat session to avoid contextual bias. The model processed images using its native multimodal capabilities without access to the raw Digital Imaging and Communications in Medicine (DICOM) metadata. 

ChatGPT image analysis

For each case, the selected CT images were uploaded to ChatGPT for analysis using a standardized prompt designed to evaluate the presence and characteristics of intracranial hemorrhage.

**Figure 1:**
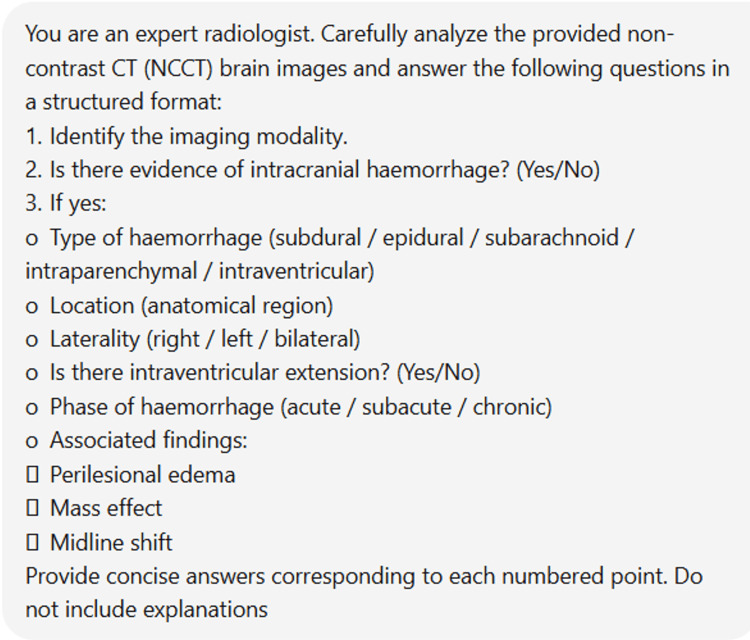
Standardized prompt used for all cases

Figure 1 shows the standardized prompt used for all cases.

The same prompt was used for all cases without modification.

Image interpretation and reference standard

Two radiologists with a minimum experience of five years independently reviewed the brain CT examinations without access to ChatGPT outputs and the responses generated by ChatGPT. In cases of discrepancy, a consensus interpretation was reached through discussion. The consensus interpretation was used as the reference standard. 

Statistical analysis

Statistical analysis was performed using IBM SPSS Statistics for Windows, V. 23.0 (IBM Corp., Armonk, NY, USA). Continuous variables were expressed as mean ± standard deviation (SD), while categorical variables were presented as numbers (n) and percentages. Gender distribution between the intracranial hemorrhage group and the control group was compared using the chi-squared test. The diagnostic performance of ChatGPT in detecting intracranial hemorrhage was evaluated by calculating sensitivity, specificity, positive predictive value (PPV), negative predictive value (NPV), and overall diagnostic accuracy, along with their 95% confidence intervals (CI). 

A chi-squared test or Fisher's exact test (where appropriate) was used to assess the association between ChatGPT predictions and radiologist diagnoses. McNemar's test was used to compare paired proportions between ChatGPT predictions and radiologist diagnoses. For chi-squared tests, degrees of freedom (df) and effect size (phi coefficient, φ) were reported.

Subtype classification was evaluated among hemorrhage-positive cases by comparing ChatGPT-predicted hemorrhage subtypes with the radiologist consensus diagnosis. For subtype-specific analysis, each hemorrhage subtype was treated as an independent binary variable. Cases with mixed hemorrhage were included in all relevant subtype categories. For subtype-specific analysis, diagnostic performance metrics including sensitivity, specificity, PPV, NPV, and accuracy were calculated separately for each hemorrhage subtype by treating each subtype as an independent binary variable.

Agreement between ChatGPT interpretations and radiologist reports was assessed using Cohen's kappa coefficient. A p-value of <0.05 was considered statistically significant.

## Results

Study population

**Figure 2:**
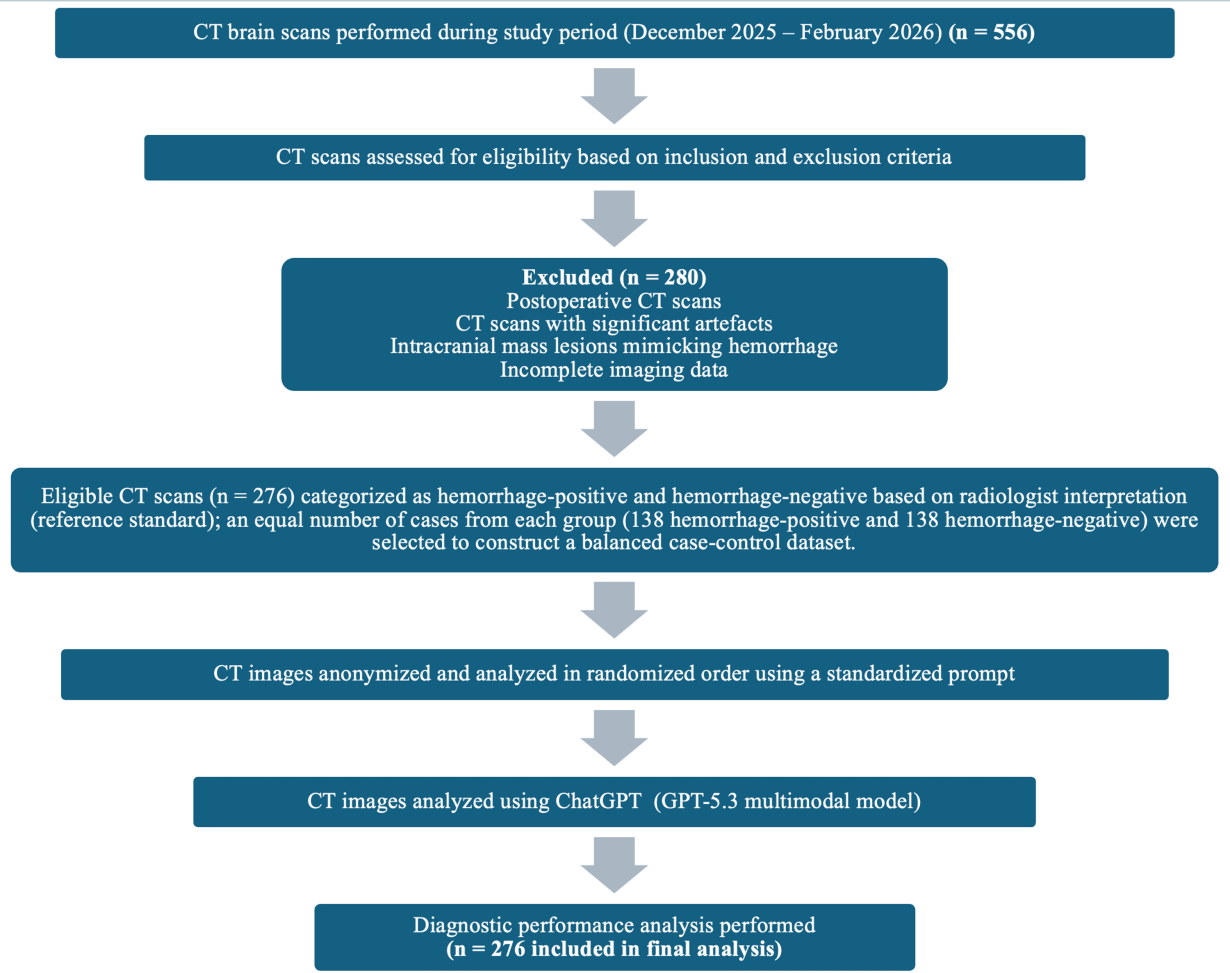
STARD flow diagram of patient selection and analysis A total of 556 CT brain scans were screened; 276 met the inclusion criteria and were categorized by radiologists as hemorrhage-positive or hemorrhage-negative. From these, 138 cases from each group were selected to form a balanced case-control dataset. ChatGPT analysis was performed and compared with the radiologist consensus. STARD: Standards for Reporting of Diagnostic Accuracy Studies

A total of 556 CT brain scans performed during the study period (December 2025 to February 2026) were screened for eligibility. After applying inclusion and exclusion criteria, 276 NCCT brain examinations were deemed eligible. These cases were categorized as hemorrhage-positive and hemorrhage-negative based on radiologist interpretation (reference standard). To construct a balanced case-control dataset, an equal number of cases from each group (138 hemorrhage-positive and 138 hemorrhage-negative) were selected for analysis from the eligible pool. ChatGPT evaluation was subsequently performed and was not involved in case selection or group allocation. The study workflow is illustrated in Figure 2.

In the hemorrhage group, 98 (71%) were male and 40 (29%) were female. In the control group, 94 (68%) were male and 44 (32%) were female. There was no statistically significant difference in gender distribution between the groups (χ² = 0.32; df = 1; p = 0.57; φ = 0.03). The gender distribution of the study population is presented in Table 1.

**Table 1:** Gender distribution of the study population Distribution of male and female patients in the ICH and control groups. No statistically significant difference in gender distribution was observed between the groups (χ² = 0.32; df = 1; p = 0.57; φ = 0.03). ICH: intracranial hemorrhage

Gender	ICH group (n = 138)	Control group (n = 138)	Total (n = 276)
Male	98 (71%)	94 (68%)	192 (69.6%)
Female	40 (29%)	44 (32%)	84 (30.4%)
Total	138 (100%)	138 (100%)	276 (100%)

Confusion matrix analysis of ChatGPT predictions

ChatGPT correctly identified 124 of 138 hemorrhage-positive cases (sensitivity: 89.9%) and 117 of 138 hemorrhage-negative cases (specificity: 84.8%). The distribution of classification outcomes is summarized in Table 2. A statistically significant association was observed between ChatGPT predictions and radiologist diagnoses (χ² = 154.1; df = 1; p < 0.001; φ = 0.75).

**Table 2:** Confusion matrix comparing ChatGPT predictions with radiologist reference standard Comparison of ChatGPT predictions with radiologist consensus interpretation for the detection of intracranial hemorrhage. A statistically significant association was observed between ChatGPT and radiologist diagnoses (χ² = 154.1; df = 1; p < 0.001; φ = 0.75), with no significant difference on McNemar's test (χ² = 1.03; p = 0.31).

	Radiologist positive	Radiologist negative	Total
ChatGPT positive	124 (true positive)	21 (false positive)	145
ChatGPT negative	14 (false negative)	117 (true negative)	131
Total	138	138	276

Diagnostic performance metrics of ChatGPT

The diagnostic performance of ChatGPT demonstrated a sensitivity of 89.9% (95% CI: 83.6-94.3) and a specificity of 84.8% (95% CI: 77.7-90.3). PPV, NPV, and overall accuracy are summarized in Table 3.

**Table 3:** Diagnostic performance metrics of ChatGPT for the detection of intracranial hemorrhage Sensitivity, specificity, PPV, NPV, and overall accuracy of ChatGPT in detecting intracranial hemorrhage, along with 95% confidence intervals. PPV: positive predictive value; NPV: negative predictive value

Parameter	Value (%)	95% confidence interval
Sensitivity	89.9	83.6-94.3
Specificity	84.8	77.7-90.3
PPV	85.5	78.6-90.8
NPV	89.3	83.0-93.9
Overall accuracy	87.3	82.8-91.0

Distribution of intracranial hemorrhage subtypes

A total of 186 components were identified among 138 cases. Intraparenchymal hemorrhage was the most common (68 (49.3%)), followed by subdural (52 (37.7%)), subarachnoid (42 (30.4%)), and epidural (24 (17.4%)) hemorrhages. Mixed involvement was observed in 48 cases. The distribution of subtypes is summarized in Table 4.

**Table 4:** Distribution of intracranial hemorrhage subtypes Distribution of hemorrhage subtypes among hemorrhage-positive cases. Values are expressed as n (%). Percentages exceed 100% as cases with mixed involvement contributed to multiple subtype categories. IPH: intraparenchymal hemorrhage; SAH: subarachnoid hemorrhage; SDH: subdural hemorrhage; EDH: epidural hemorrhage

Hemorrhage type	Cases (n)	Percentage (%)
IPH	68	49.30%
SAH	42	30.40%
SDH	52	37.70%
EDH	24	17.40%

Subtype-specific diagnostic performance of ChatGPT

Subtype-specific diagnostic performance metrics were calculated for each hemorrhage subtype and are summarized in Table 5.

**Table 5:** Subtype-specific diagnostic performance of ChatGPT for intracranial hemorrhage Diagnostic performance metrics for individual hemorrhage subtypes, including sensitivity, specificity, PPV, NPV, and accuracy. Each subtype was analyzed as an independent binary variable. Cases with mixed involvement contributed to all relevant subtype categories. IPH: intraparenchymal hemorrhage; SAH: subarachnoid hemorrhage; SDH: subdural hemorrhage; EDH: epidural hemorrhage; TP: true positive; FP: false positive; FN: false negative; TN: true negative; PPV: positive predictive value; NPV: negative predictive value

Hemorrhage subtype	Total cases (n)	TP	FN	FP	TN	Sensitivity (%)	Specificity (%)	PPV (%)	NPV (%)	Accuracy (%)
IPH	68	60	8	10	198	88.2	95.2	85.7	96.1	93.5
SAH	42	31	11	21	213	73.8	91	59.6	95.1	88.4
SDH	52	32	20	24	200	61.5	89.3	57.1	90.9	84.1
EDH	24	16	8	19	233	66.7	92.5	45.7	96.7	90.2

**Figure 3:**
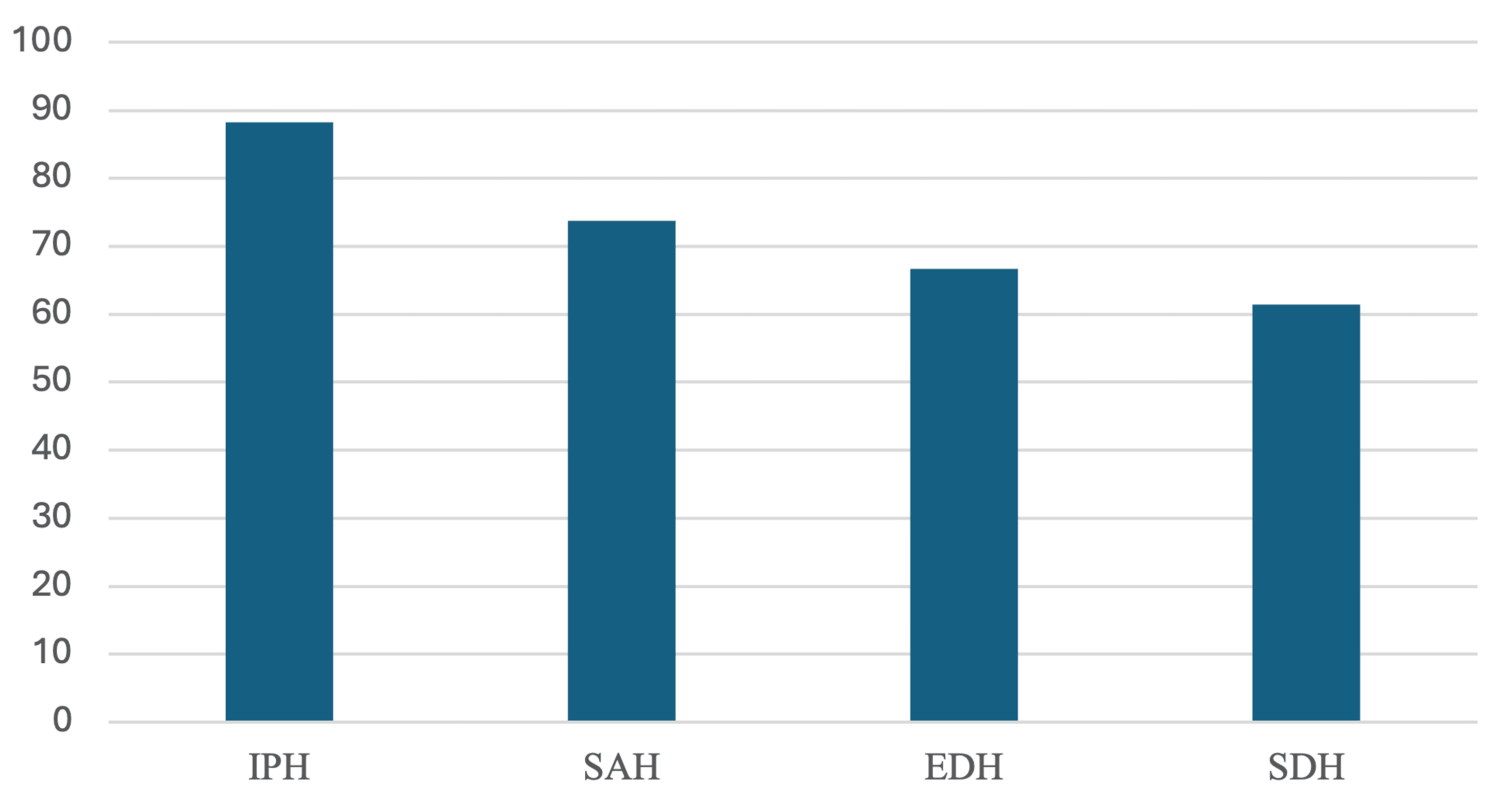
Subtype-specific sensitivity of ChatGPT for the detection of intracranial hemorrhage Bar chart illustrating the sensitivity of ChatGPT in detecting different intracranial hemorrhage subtypes, including IPH, SAH, EDH, and SDH. IPH: intraparenchymal hemorrhage; SAH: subarachnoid hemorrhage; SDH: subdural hemorrhage; EDH: epidural hemorrhage

The highest detection sensitivity was observed for intraparenchymal hemorrhage (88.2%), followed by subarachnoid (73.8%), epidural (66.7%), and subdural (61.5%) hemorrhages. Sensitivity across hemorrhage subtypes is illustrated in Figure 3.

Accuracy of additional imaging feature characterization

The accuracy of ChatGPT in characterizing additional imaging features, including hemorrhage location and laterality, is summarized in Table 6.

**Table 6:** Accuracy of ChatGPT in the characterization of additional imaging features Component-wise evaluation of ChatGPT performance in identifying hemorrhage location and laterality. Each hemorrhage component was analyzed independently. Values are expressed as numbers (n) and percentages.

Feature	Correct (n)	Incorrect (n)	Accuracy (%)
Location	146	40	78.50%
Laterality	133	53	71.60%

Agreement analysis

Agreement between ChatGPT interpretations and radiologist reports was assessed using Cohen's kappa coefficient, calculated from the confusion matrix for hemorrhage detection, demonstrating good agreement (κ = 0.75).

Representative imaging examples

**Figure 4:**
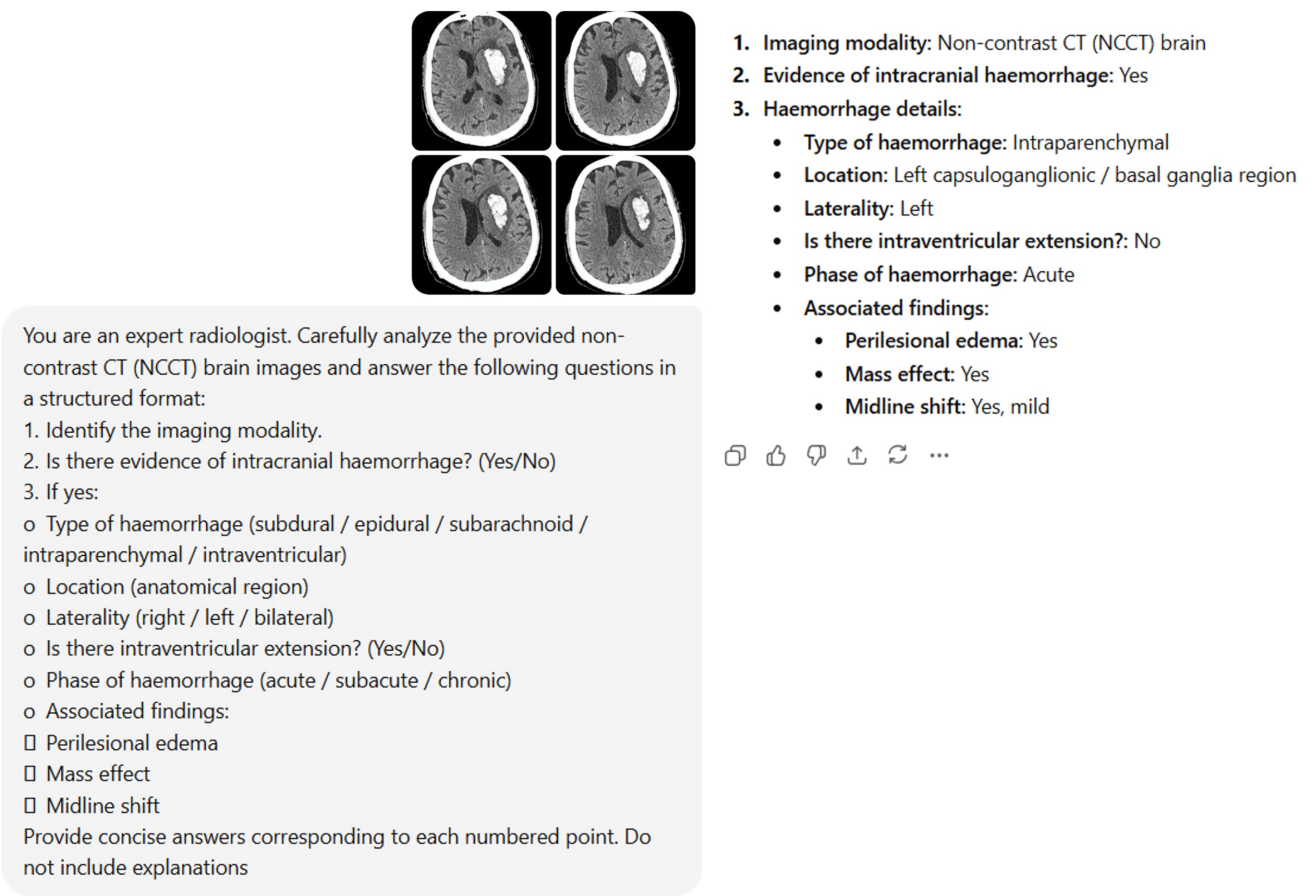
Representative NCCT brain image correctly identified by ChatGPT showing left gangliocapsular intraparenchymal hemorrhage Axial NCCT images of the brain demonstrate a hyperdense intraparenchymal hemorrhage in the left gangliocapsular (basal ganglia) region, with associated perilesional edema and mild mass effect. The findings were correctly identified by ChatGPT and are concordant with the radiologist interpretation. NCCT: non-contrast computed tomography

**Figure 5:**
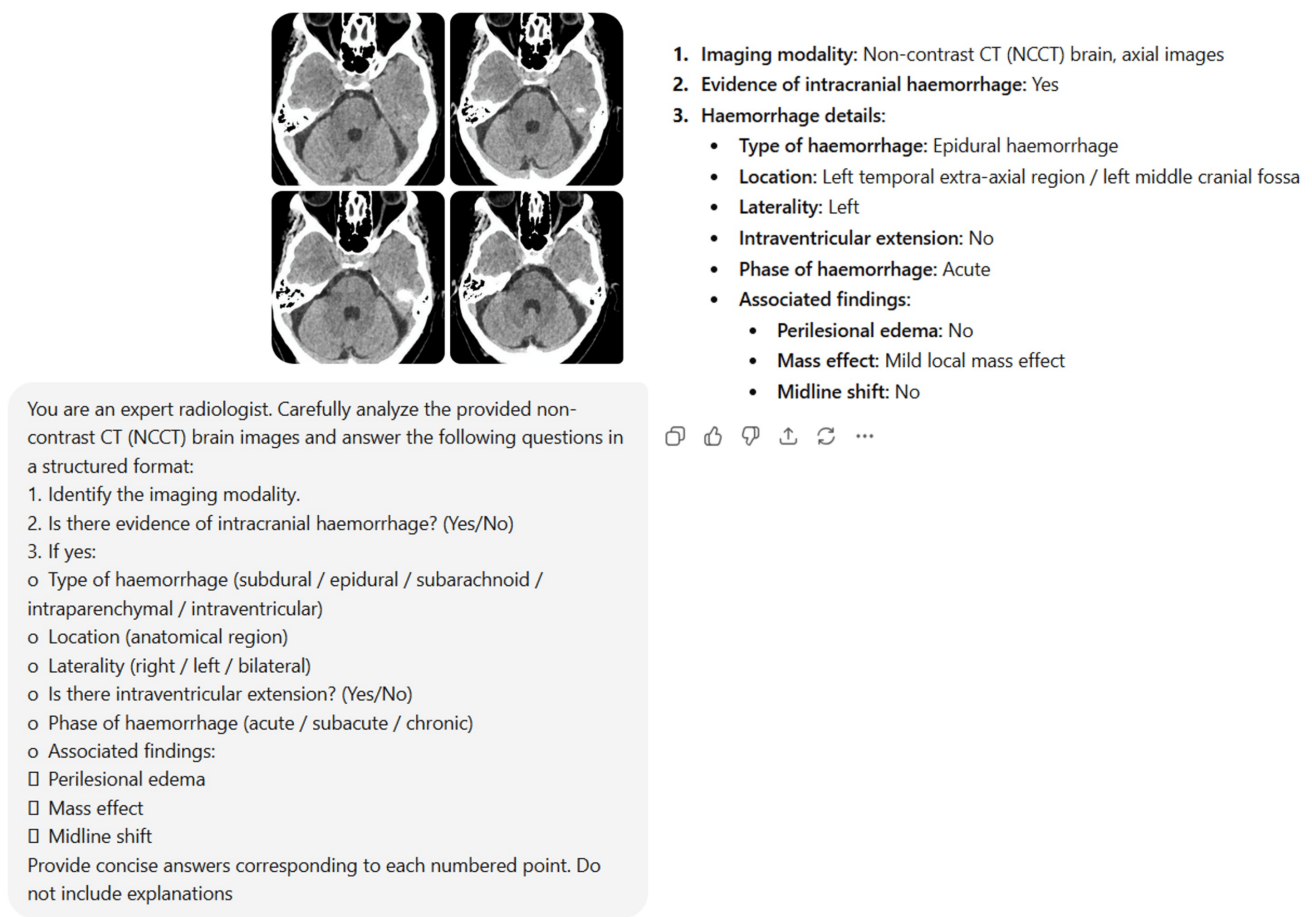
Representative NCCT brain image incorrectly interpreted by ChatGPT as hemorrhage Axial NCCT images of the brain show no evidence of intracranial hemorrhage. However, ChatGPT incorrectly identified the images as demonstrating epidural hemorrhage along the left temporal region, representing a false-positive interpretation. NCCT: non-contrast computed tomography

Representative NCCT brain images demonstrating correct and incorrect ChatGPT interpretations are shown in Figure 4 and Figure 5.

## Discussion

In the present study, we evaluated the diagnostic performance of ChatGPT in detecting intracranial hemorrhage on NCCT brain scans. Radiologist interpretation served as the reference standard for classification prior to ChatGPT evaluation. The results demonstrated a sensitivity of 89.9% and specificity of 84.8%, indicating that ChatGPT correctly identifies the majority of hemorrhage cases but may produce false-positive results in certain instances. Agreement analysis showed good concordance between ChatGPT and radiologist interpretations, with a Cohen's kappa value of 0.75.

These findings are similar to those reported by Koyun et al. who evaluated ChatGPT-4o for hemorrhage detection and reported moderate diagnostic performance (sensitivity 79.2%, specificity 57.5%) [11]. Similarly, Zhang et al. demonstrated the potential of GPT-4 in identifying cerebral hemorrhage on CT scans [10]. In our study, ChatGPT demonstrated moderate diagnostic performance with variable sensitivity across hemorrhage subtypes. It should be noted that the prompt used in the present study was not identical to those used in prior studies, although a similar structured approach for radiological assessment was employed. Variations in prompt design may influence model performance; therefore, direct comparisons should be interpreted with caution.

Although these findings are promising, deep learning algorithms made specifically for detecting intracranial hemorrhage usually perform better. Wang et al. created a deep learning algorithm that can automatically detect and classify acute intracranial hemorrhage on head CT scans with high accuracy [4]. Other studies have also shown that AI-based models have demonstrated high accuracy in detecting intracranial hemorrhage and improving diagnostic workflows in clinical practice [3,5,12,13].

Recent research highlights the expanding role of AI in neuroimaging and clinical decision support. Studies show deep learning models effectively detect intracranial abnormalities and improve diagnostic efficiency in radiology [14]. Broader reviews emphasize AI's growing integration in neuroimaging workflows and its potential to enhance diagnostic accuracy and clinical outcomes [15].

In our study, ChatGPT showed the highest subtype sensitivity for intraparenchymal hemorrhage, followed by subarachnoid, epidural, and subdural hemorrhages. The higher sensitivity for intraparenchymal hemorrhage may be attributed to its conspicuous hyperdensity and well-defined margins, whereas extra-axial hemorrhages such as subdural and epidural types may be more subtle and challenging to detect.

ChatGPT demonstrated moderate accuracy in identifying additional imaging features such as hemorrhage location and laterality, indicating reasonable spatial interpretation capability, although with some limitations in precise anatomical localization.

Despite good overall performance, certain limitations in hemorrhage detection were observed. False-positive results may be related to imaging artifacts such as beam hardening, which can mimic hyperdense lesions. False-negative cases likely reflect difficulty in detecting subtle hemorrhages, particularly thin subdural collections, which often require optimized window settings such as subdural windows.

The use of selected image slices rather than the full CT dataset may have contributed to missed detections and may have overrepresented hemorrhagic regions, potentially leading to an overestimation of model performance. Furthermore, conversion of CT images from the console/PACS to standard image formats (PNG/JPEG) may introduce variability in image quality and contrast representation, potentially affecting model performance. 

The use of a case-control design with an artificially balanced dataset (equal proportions of hemorrhage-positive and hemorrhage-negative cases) may not reflect real-world disease prevalence in emergency settings. This may influence predictive values (PPV and NPV) and limit the generalizability of the findings. Future studies using full volumetric datasets and prevalence-based cohorts are warranted to better reflect real-world clinical performance. Inter-observer agreement between radiologists prior to consensus was not quantified, and the number of discordant cases was not recorded.

While ChatGPT is not specifically trained for medical imaging, its ability to identify hemorrhages in many cases suggests it could support radiology workflows, including preliminary screening and triage. ChatGPT should not be regarded as a substitute for expert radiologist interpretation; rather, it may serve as an adjunctive tool. 

## Conclusions

ChatGPT demonstrated high sensitivity and specificity in detecting intracranial hemorrhage on NCCT brain scans. While it cannot replace radiologist interpretation, ChatGPT, as an LLM, may serve as an adjunct tool for preliminary screening and triage. Further studies with larger datasets and model optimization are required before routine clinical use.
